# Toxigenic profile of methicillin-sensitive and resistant *Staphylococcus aureus* isolated from special groups

**DOI:** 10.1186/s12941-016-0125-5

**Published:** 2016-02-16

**Authors:** Camila Sena Martins de Souza, Carlos Magno Castelo Branco Fortaleza, Claudia Lima Witzel, Mônica Silveira, Mariana Fávero Bonesso, Silvio Alencar Marques, Maria de Lourdes Ribeiro de Souza da Cunha

**Affiliations:** Department of Microbiology and Immunology, Botucatu Biosciences Institute, UNESP-Univ Estadual Paulista, Botucatu, SP Brazil; Department of Tropical Diseases, Botucatu School of Medicine, University Hospital, UNESP-Univ Estadual Paulista, Botucatu, SP Brazil; Department of Dermatology and Radiology, Botucatu School of Medicine, University Hospital, UNESP-Univ Estadual Paulista, Botucatu, SP Brazil

**Keywords:** *Staphylococcus aureus*, MSSA, Toxins, Cytotoxins

## Abstract

**Background:**

*Staphylococcus aureus* is characterized by its pathogenicity and high prevalence, causing disease in both healthy and immunocompromised individuals due to its easy dissemination. This fact is aggravated by the widespread dissemination of *S. aureus* carrying toxigenic genes. The objective of this study was to determine the toxigenic profile of methicillin-sensitive *S. aureus* (MSSA) and methicillin-resistant *S. aureus* (MRSA) in patients with purulent skin and/or soft tissue infections seen at the Dermatology Department of the University Hospital of the Botucatu Medical School, asymptomatic adults older than 60 years living in nursing homes, and prison inmates of the Avaré Detention Center.

**Methods:**

PCR was used for the detection of the *mecA* gene, enterotoxin genes (*sea*, *seb*, and *sec*), exfoliative toxins A and B (*eta* and *etb*), toxic shock syndrome toxin 1 (*tst*), panton–valentine leukocidin (*lukS*-*PV* and *lukF*-*PV*), and alpha- and delta-hemolysins or cytotoxins (*hla* and *hld*).

**Results:**

The results showed a significant prevalence of toxigenic genes among *S. aureus* isolates from asymptomatic individuals, with the observation of a higher prevalence of cytotoxin genes. However, the panton–valentine leukocidin gene was only detected in MSSA isolated from patients with skin infections and the *tst* gene was exclusively found in MSSA isolated from prison inmates.

**Conclusions:**

The present study demonstrated a significant prevalence of toxigenic genes in MSSA and MRSA strains isolated from asymptomatic *S. aureus* carriers. There was a higher prevalence of cytotoxin genes.

## Background

*Staphylococcus aureus* is one of the pathogens most frequently isolated from nosocomial and community-acquired infections and represents a major public health problem because it easily acquires resistance to commonly used antibiotics. Skin and soft tissue infections (SSTI) are common bacterial infections in humans and treatment is difficult because of the increasing frequency of methicillin-resistant *S. aureus* (MRSA) in the community [1, 2].

Nursing homes have been recognized as reservoirs of *S. aureus*. This fact is a consequence of the advanced age of elderly people, lifestyle, need for invasive devices, presence of chronic wounds, dependence on healthcare workers, and previous hospitalization. Through direct contact, MRSA colonization within nursing homes results in spread to other residents, relatives, and healthcare workers. In addition, the elderly population represents a vulnerable group that is often hospitalized. This leads to the dissemination of the microorganism within hospitals, which renders control measures in the open community ineffective [3, 4].

SSTI secondary to *S. aureus* are notorious in the prison setting. The sharing of personal items and lack of hygiene within this crowded environment facilitates the propagation and transmission of *S. aureus* between inmates and their visitors from the community [5].

After entering the host, infection develops when a pathogen escapes recognition and destruction by the host’s immune system. For this purpose, *S. aureus* expresses different virulence factors that promote survival and pathogenesis within a host [6]. Methicillin-sensitive *S. aureus* (MSSA) plays an important role by carrying virulence factors that increase its capacity for invasion and survival. In addition, MSSA has been suggested to give origin to MRSA strains by acquiring the *mecA* gene from coagulase-negative staphylococci found on the skin of healthy individuals.

The objective of this study was to determine the toxigenic profile of MSSA and MRSA isolated from nursing home residents, prison inmates, and patients with purulent SSTI.

## Experimental section

### Strains

A total of 150 *S. aureus* strains were analyzed. The samples included in this study were obtained from three previous studies aimed at determining the prevalence of *S. aureus* [24–26]. Fifty *S. aureus* strains were isolated from 302 prison inmates between February 2009 and March 2010 [24]. The inclusion criterion was a minimum prison stay of 3 months. The study excluded individuals who utilized antibiotics during 30 days prior to sample collection. Also excluded were patients who were admitted to a hospital and those that underwent invasive procedure in the year preceding sample collection.

In addition, 50 *S. aureus* strains isolated from patients with purulent SSTI treated at the Dermatology outpatient clinic of the University Hospital of the Botucatu Medical School (HC-FMB), São Paulo, Brazil, between 2008 and 2009 were included. Exclusion criteria were the same in the study with inmates. The prevalence study [25] included 127 patients from which 66 (56.9 %) *S. aureus* isolates were obtained. However, since the aim of this study was to compare the virulence of *S. aureus* from different populations, we selected randomly 50 isolates to match the number of strains isolated from inmates.

The final 50 strains were randomly selected among 52 *S. aureus* strains isolated from 300 older adults living in seven nursing homes in Bauru, São Paulo, Brazil, in 2011 and 2012 [26]. All adults older than 60 years who agreed to participate in the study were included, regardless of their health status. Institutionalized individuals with less than 30 days were excluded.

### Phenotypic and genotypic identification

The swabs were seeded on Baird-Parker agar and typical colonies were submitted to Gram staining for analysis of purity and observation of colony morphology. Catalase, coagulase and additional biochemical tests (trehalose, maltose, and mannitol) were used for the identification of *S. aureus* [15, 16].

The isolates were also submitted to genotypic identification using the SA442 primer, which is specific for *S. aureus* [17].

### Detection of methicillin resistance

The *mecA* gene was detected by the polymerase chain reaction (PCR) [18]. International references strains were included in all reactions as positive (*S. aureus* ATCC 33591) and negative controls (*S. aureus* ATCC 25923).

### Detection of toxin genes

PCR was used to detect genes that encode the following toxins: classical enterotoxins (*sea*, *seb*, and *sec*-1) [19, 20], panton–valentine leukocidin (PVL) (*luk*PV) [21], toxic shock syndrome toxin 1 (*tst*) [19, 20], exfoliative toxins A and B (*eta* and *etb*) [22], and cytotoxins (*hla* and *hld*) [23].

### Visualization of amplified products

The efficiency of amplification was evaluated by electrophoresis on 2 % agarose gel prepared in 1× TBE buffer and stained with SYBR Safe. The size of the amplified product was compared to a 100-bp standard and the gel was photographed under UV transillumination.

## Results

**Fig. 1:**
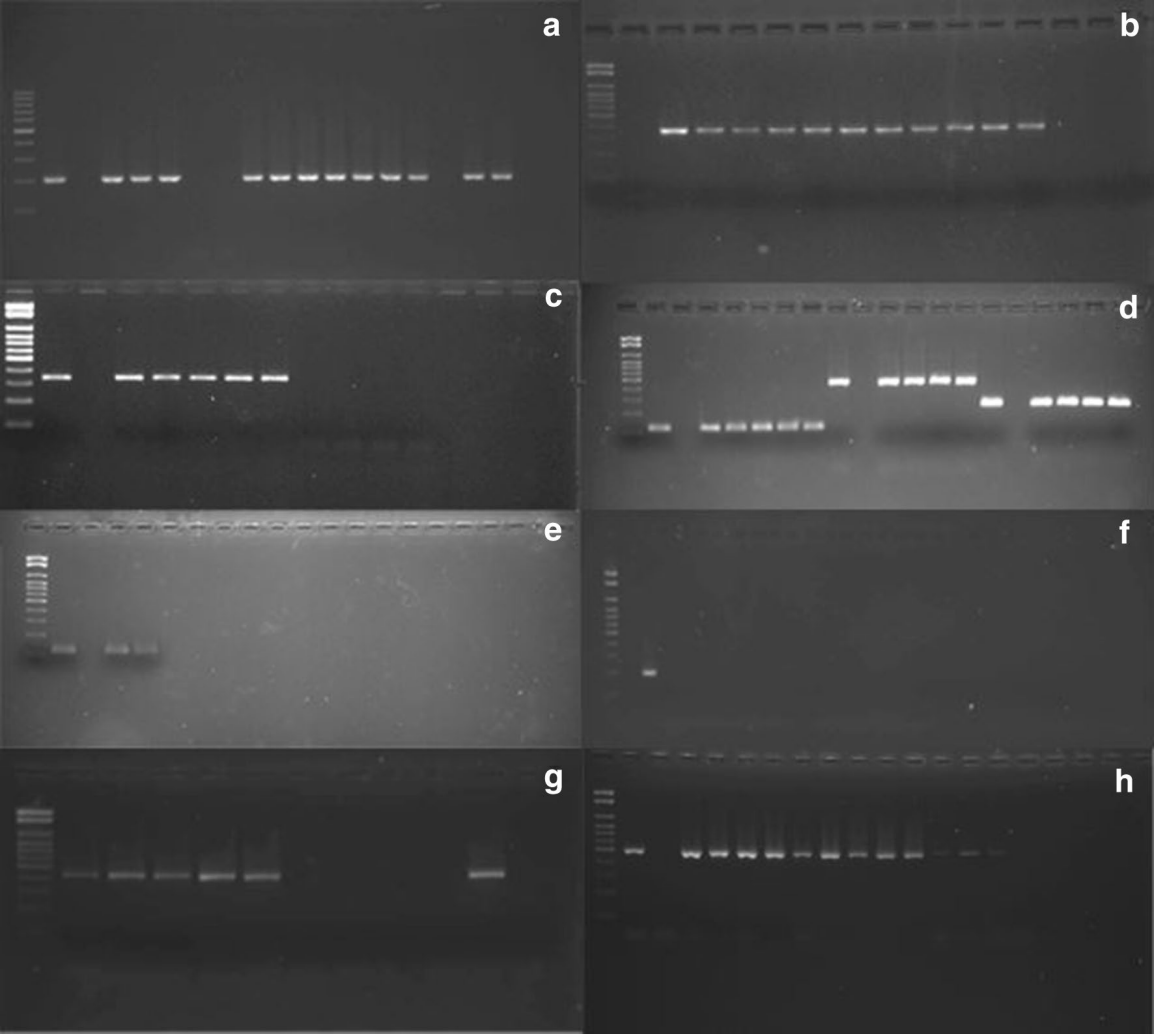
Agarose gel electrophoresis (stained with SYBR^®^ safe) showing the amplified products positive controls, negative and some samples studied. Gel A: *hla* (209pb), Gel B: *hld* (357pb), Gel C: *tst* (350pb), Gel D: *sea* (120pb), *seb* (478pb), *sec*-1 (257pb), Gel E: *eta* (pb 119), Gel F: *etb* (200pb), Gel G: *pvl* (433pb), Gel H: *mec*A (533pb)

Among the 150 *S. aureus* strains studied, 20 (13.3 %) were carriers of the *mecA* gene. Of these, 11 samples were isolated from nursing home residents, while 2 were obtained from inmates. The remaining 7 were isolated from patients with SSTI. The results of all genes encoding various toxins within each subgroup are summarized in Table 1. Sixty-three isolates (35.3 %) did not carry any of the enterotoxin genes, while 87 (58 %) carried at least one gene. Three cases notably harbored three enterotoxin genes (one collected from the Dermatology clinic versus two obtained from inmates). No differences in cytotoxin genes were observed between MRSA and MSSA isolates. The genes encoding PVL were evidently restricted to isolates obtained from the subgroup of patients with purulent SSTI. Some of the amplified genes can be observed in Fig. 1.

**Table 1:** Investigation of genes encoding superantigens in the MRSA and MSSA isolates

Origin	Enterotoxin			Exfoliatin		TSST-1	Hemolysin		PVL
	sea n (%)	seb n (%)	sec-1 n (%)	eta n (%)	etb n (%)	tst	hla n (%)	hld n (%)	pvl n (%)
Nursing home									
MRSA (n = 11)	6 (54.5)	1 (9)	0	0	0	0	11 (100)	11 (100)	0
MSSA (n = 39)	16 (41)	14 (36)	2 (5.1)	0	0	0	39 (100)	39 (100)	0
CR									
MRSA (n = 2)	0	0	1	0	0	0	2 (100)	2 (100)	0
MSSA (n = 48)	16 (33.3)	13 (27)	7 (14.6)	2 (4.1)	0	5 (10.4)	47 (97.9)	47 (97.9)	0
Dermatology									
MRSA (n = 7)	2 (28.5)	1 (14.2)	0	0	0	0	6 (85.7)	5 (71.4)	0
MSSA (n = 43)	16 (37.2)	9 (21)	8 (18.6)	1 (2.3)	0	0	39 (90.7)	39 (90.7)	10 (23.6)

*CR* Centro de Ressocialização de Avaré (Detention Center), *MRSA* methicillin-resistant *Staphylococcus aureus*, *MSSA* methicillin-sensitive *Staphylococcus aureus*, *TSST*-*1* toxic shock syndrome toxin 1, *PVL* panton–valentine leukocidin

## Discussion

The genotypic toxigenic profile of the isolates studied differed according to their origin. The results showed a higher prevalence of the enterotoxin A gene in all strains studied. Previous studies also reported a higher prevalence of the *sea* gene compared to the other enterotoxin genes in *S. aureus* isolated from hospitalized patients [7, 8]. However, the higher prevalence of this gene in *S. aureus* isolated from healthy subjects is a new finding. Furthermore, 10 % (n = 5) of the isolates from the Detention Center harbored the toxic shock syndrome toxin 1 gene, whereas this gene was not detected in any of the isolates from the nursing homes or Dermatology clinic. The presence of these super antigen genes in MRSA and MSSA isolated from patients and healthy subjects implies the possibility of increased bacterial dissemination and more severe infections, since these toxins induce the activation of T cells and exacerbated secretion of cytokines, causing episodes of severe sepsis [9].

Different virulence factors are implicated in the pathogenesis of *S. aureus*, including exfoliatins. The *eta* gene was detected in 4 % (n = 2) of isolates from the Detention Center and in only one patient from the Dermatology clinic. The latter patient had bullous impetigo at the time of sample collection, indicating a possible expression of the *eta* gene by the bacterium. However, the *etb* gene was not detected in any isolate. This finding agrees with another study in which MRSA and MSSA isolated from a tertiary hospital did not harbor the exfoliatin B gene [8].

Colonization with *S. aureus* is a characteristic feature of several inflammatory skin diseases, which is often followed by epidermal damage and invasive infection. This damage is the result of the dermonecrotic activity of cytotoxins [10]. Alpha-cytotoxin acts by forming pores in the target membrane and delta-cytotoxin acts as a surfactant. The two cytotoxins or hemolysins are involved in erythrocyte lysis [11]. The present results demonstrate a high prevalence of alpha- and delta-cytotoxin genes in MSSA and MRSA isolates of all origins. Therefore, hemolytic activity is paramount to an organism’s virulence and its capacity for pathogenesis since the resulting degradation of host tissue permits invasion, dissemination, and escape from the immune system [12].

Investigation of the *pvl* gene revealed the presence of *pvl* gene-positive *S. aureus* only in patients of the Dermatology clinic (n = 10, 20 %). These strains were isolated from primary and secondary infections. A study in the United Kingdom that examined 65 nursing homes similarly did not detect the *pvl* gene. However, in the present study, none of the MRSA isolates carried the *pvl* gene, thus refuting the suggestion that this gene is a good marker of MRSA strains [4]. Our results are in agreement with previous studies that have demonstrated lysogeny of MSSA a phage that harbors *pvl* genes and subsequently acquires the *mecA* gene [13]. Other studies have similarly identified MRSA strains that lacked genes encoding *pvl* [14].

A strength of this study is the comparison of the presence of several genes encoding virulence factors (enterotoxins, exfoliatins, TSST-1, cytotoxins, and PVL) between MSSA and MRSA from different populations. However, a limitation was the fact that only the presence of these genes was investigated. Future studies evaluating the expression of these virulence factors are therefore important. Knowledge of the pathogenic profile of *S. aureus* permits a better understanding and control of this microorganism, which has become one of the main causative agents of healthcare-associated infections.

The present study demonstrated a significant prevalence of toxigenic genes in MSSA and MRSA strains isolated from asymptomatic *S. aureus* carriers, with the observation of a higher prevalence of cytotoxin genes. These results are important and indicate the need for further case–control or cohort studies to determine the role of these toxins in the occurrence of staphylococcal infections.

### Availability of supporting data

The data set supporting the results of this article is included in the article.

## Abbreviations

MRSA: methicillin-resistant *Staphylococcus aureus*
*mecA*: methicillin resistance gene
*sea*: staphylococcal enterotoxin A
*seb*: staphylococcal enterotoxin B
*sec*: staphylococcal enterotoxin C
*eta*: exfoliative toxin A
*etb*: exfoliative toxin B
*luk*-*PV*: panton–valentine leukocidin
*tst*: toxic shock syndrome toxin 1
*hla*: alpha-hemolysin
*hld*: delta-hemolysin
PCR: polymerase chain reaction
HCFMB: University Hospital of the Botucatu Medical School
CR: detention center
